# Lytic bone lesion of the skull as a rare manifestation of hepatocellular carcinoma: a case report

**DOI:** 10.1186/s13037-025-00434-2

**Published:** 2025-04-22

**Authors:** Sherif Wael, Omar Hamdy, Mohamed Yasser, Sara Elmandrawi, Mai Mostafa, Nouran Mohammed, Ahmed Elghrieb

**Affiliations:** 1 Mansoura University Hospitals, Mansoura, Egypt; 2 Surgical Oncology Department, Mansoura University Oncology Center, Mansoura, Egypt; 3 Department of General Surgery, Faculty of Medicine, Mansoura University, Mansoura, Egypt

**Keywords:** Hepatocellular carcinoma, Skull metastasis, Hepatitis C, Neurosurgery, Solitary bone metastasis

## Abstract

**Background:**

Hepatocellular carcinoma (HCC) ranks among the leading causes of cancer-related deaths worldwide, with metastatic spread to bones being alarmingly frequent. However, HCC metastases to the skull are notably rare, accounting for only 0.4–1.6% of all bone metastases. Typically, metastases are found in the spine, pelvis, and ribs. The occurrence of solitary skull metastases, especially in the absence of active primary liver cancer, is extremely uncommon.

**Case description:**

We present the clinical case of a 57-year-old male patient with a documented history of hepatitis C virus infection but without prior evidence of active hepatocellular carcinoma. Over the course of several months, he developed a non-tender, progressively enlarging mass located in the occipital region of the skull. A computed tomography (CT) scan identified a lytic lesion with intracranial compression, although no midline shift was noted. Histopathological examination confirmed the lesion as metastatic HCC, further supported by immunohistochemical markers Hepatari- 1 and Cytokeratin- 19. Subsequent diagnostic procedures revealed hepatic lesions, including a positron emission tomography (PET)-CT scan. Further examination through CT imaging of the abdomen with contrast highlighted a well-defined focal lesion in hepatic segment 4a, measuring 4.3 × 4.3 cm, predominantly enhancing with HCC characteristics. The skull lesion was surgically removed en bloc, and the patient underwent adjunct radiotherapy and systemic therapy, with palliative therapy till his death in May 2024. To better understand and manage this atypical presentation, we conducted a review for the discussion of clinical manifestations, imaging findings, pathological features, and patient outcomes associated with HCC skull metastases.

**Conclusion:**

This case emphasizes the critical importance of considering hepatocellular carcinoma in the differential diagnosis of solitary skull lesions, especially in patients with risk factors for liver disease. Prompt identification of the primary malignancy remains essential for ensuring optimal management and improving patient prognosis.

## Background

Hepatocellular carcinoma (HCC) is the sixth most common cancer globally and the second leading cause of cancer-related death, primarily due to its late diagnosis and aggressive progression [1, 2]. The prognosis of HCC remains poor because most patients are diagnosed at advanced stages, where the cancer has already metastasized to distant organs [3]. Hepatocellular carcinoma metastasizes mainly to the lungs, regional lymph nodes, and bones that mostly go to the spine, pelvis, ribs, and femur [4]. It also metastasizes to the skull but in infrequent occasions like this case with about 0.4–1.6% compared to other HCC metastases [3]. According to a literature review by Jiang et al. in 2014, only 14 out of 59 reported cases of HCC-related skull metastases involved a solitary lesion, with just five of these cases initially presenting without a known primary cancer [5]. Skull lesions may be mistaken for benign conditions such as cysts or sebaceous tumors, delaying the identification of the metastatic disease, leading to the aggravation of the nature of HCC leading to a high mortality rate. This case report highlights the uncommon presentation of solitary skull metastasis from HCC. It underscores the importance of considering metastatic disease in patients with liver history, even when the clinical findings suggest benign pathology. It also draws attention to the diagnostic and therapeutic challenges associated with HCC metastasis to unusual sites like the skull, a scenario that requires careful attention to detail for accurate diagnosis and optimal management.

## Case presentation

For the case report, the patient was identified by the senior author when presenting to the clinic. No personal identifiers are disclosed and the write-up of this patient's case is by the corresponding institution’s IRB and ethical guidelines. The patient’s clinical history, presentation, and management are discussed. For the review of the literature, PubMed was used. Search terms included: “Hepatocellular carcinoma,” “HCC metastasis,” “HCC skull metastasis,” and “skull metastasis,” “solitary skull metastasis.” The resulting search results were manually filtered by authors to include articles with a title or abstract that only featured both HCC and solitary skull metastasis. Seven articles were found and are highlighted.

The patient is 57-year-old male with a positive history of hepatitis C virus since 2015 with no history of active HCC lesion, who began to grow a non-tender skull mass in November 2021. He did not seek care until January 2022, when he presented due to gradual enlargement of the skull mass. On general examination, the patient appeared well with normal complexion and all vital signs were within normal range. With neurological and local examination of the mass, the patient was neurologically intact with no skin stigmata. The mass was measured approximately 7 × 2 cm and protruded about 3 cm above the scalp surface. Both scalp and hair overlying the lesion were normal except for the stretching over the underlying lesion. The patient didn't have any associated lymphadenopathy. His oncology history was free with no chemical or radiological treatment and liver lesions which made the suspicion of metastatic mass far away from being considered. The decision was made to surgically remove the lesion as at that time there was no suspicion of being a malignant lesion. However, during the operation and while removing the sac, it was noticed that the wall covering the cyst was well-defined and strongly attached to the skull bone with no plane between the cyst and the skull, and during the procedure, the skull seemed to be degenerated and destructed. The mass was removed and well debridement was done to the surrounding bones carefully to avoid damage to the dura and the brain tissue then the specimen and the mass were sent for histopathological examination and the skin was closed primarily.

**Fig. 1:**
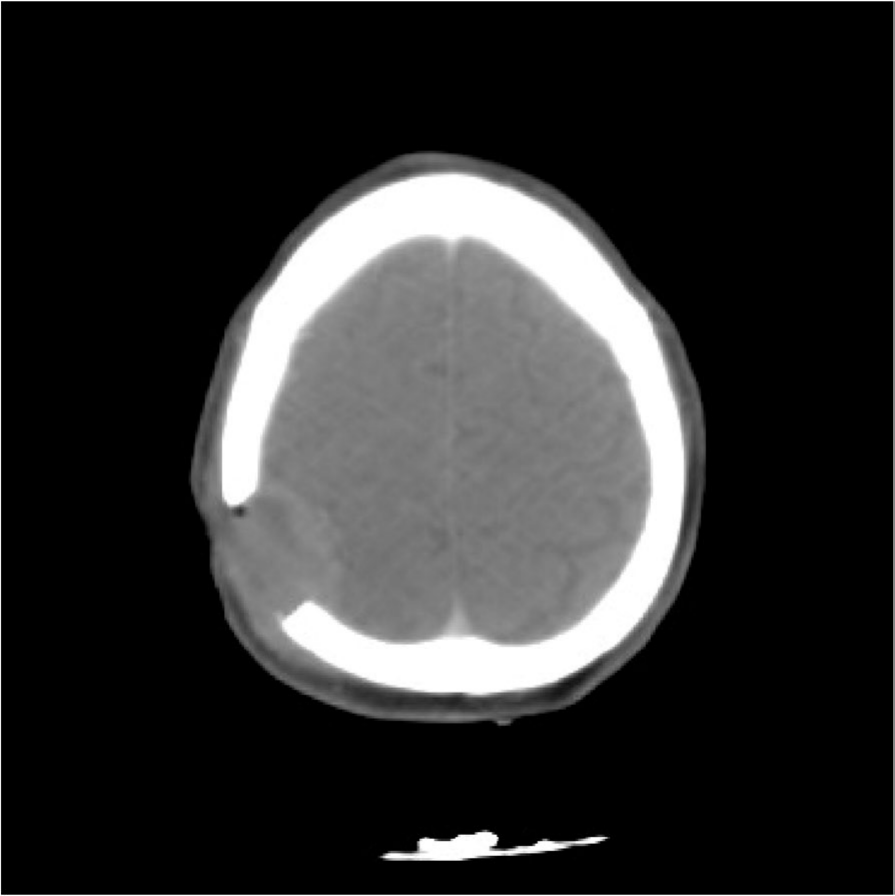
A non-contrast Ct brain showing a right occipital lytic lesion

A non -contrast multi- slice computed tomography (CT) of the brain was done showing:

Right occipital lytic lesion 5.3 * 4.9 cm, with underlying erosion of skull vault bones compressing brain parenchyma, bulging outside the skull representing metastatic deposits, no focal cerebral area of abnormal density seen, preserved normal gray – white matter interface, no extra-axial or intra-cerebral areas of fresh blood densities, normal size and configuration of the ventricular system, no midline shift, unremarkable posterior fossa structures, (Fig. 1).

First, pathological analysis of the lesion was done in 2/2022 showing the presence of ulceration of the epidermis, the dermis & subcutaneous tissue show diffuse sheets of malignant sebaceous cells centered by excess necrosis, and cells are pleomorphic with irregular nuclei showing irregular mitosis & vacuolated cytoplasm, the tumor infiltrated the underlying bone & soft tissue with no safety margins, no lymph-vascular emboli giving the result of a picture compatible with sebaceous carcinoma for Epithelial membrane antigen (EMA), Cytokeratins- 7 (CK- 7), Cytokeratins–19 (CK- 19) & androgen receptors to confirm and exclude others. The pathological analysis by the immune system-chemical method was done in 3/2022 with the use of: Epithelial membrane antigen (EMA), Cytokeratins- 7 (CK- 7), Cytokeratins–19 (CK- 19), Heppar – 1 and CD – 15 immune stains, showing:

EMA: Revealed positive membrane staining of tumor cells, CK- 7: Revealed negative cytoplasmic staining of tumor cells, CK- 19: Revealed positive cytoplasmic staining tumor cells, Heppar- 1: Revealed positive granular cytoplasmic staining of tumor cells, CD- 15: Revealed negative membrane staining of tumor cells, which gave the diagnosis of hepatocellular carcinoma that metastasized to the skull and appeared as primary symptom.

A PET CT was done in 2/2022 showing:

LOCOREGIONAL metabolically active residual operative bed 2 stmS, SUV19, LARGEST at skull: 30 * 16 * 17 mm/smaller at dura 20 * 12 * 11 mm which gives a hint for metastatic cancer, metabolic active multiple HFLs at both liver lobes invading PV, segment II, III, IV, SUV 9.3, metastatic metabolic active at LT Para-aortic lns 23 mm, SUV 12.3, metastatic metabolic active LT suprarenal gland thickening 27 * 8 mm, SUV 3.6, the possibility of synchronous double malignancy (Sebaceous carcinoma + HCC).

At this time Labs investigations were done showing normal levels of liver and renal functions but with a high level of Alpha-photo protein (AFP) which suggests a high incidence of hepatocellular carcinoma. According to these results, the patient started with Sorafenib for 6 months (started 3/2022) with disease progression that developed right occipital skull metastasis that underwent metastasectomy, then received radiotherapy 15 fx in (6/2022), then shifted to Stivarga which stopped at (3/2023).

The Child score was assisted to be found: B7, so the patient started Opdivo 240 mg IV q2 weeks, with further investigations and the general condition of the patient he was not fit for Atezolizumab plus Bevacizumab.

**Fig. 2:**
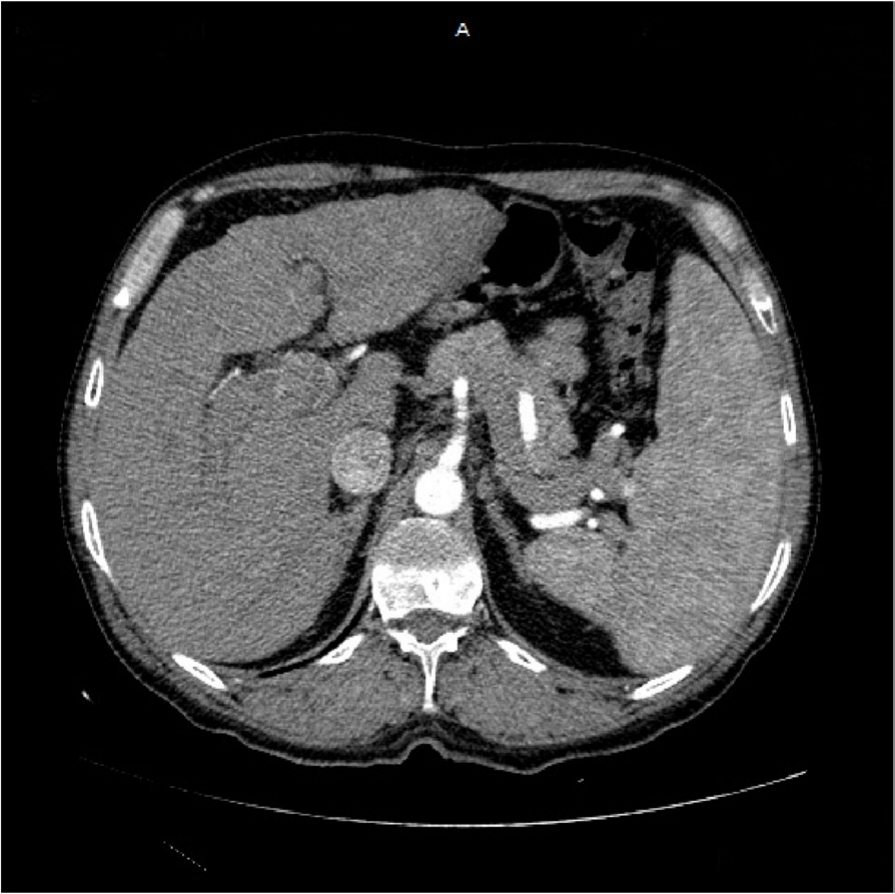
Arterial phase of CT abdomen scan showing fairly defined focal lesion in left lobe segment II measures 13.0 × 7.3 cm show diffuse enhancement

**Fig. 3:**
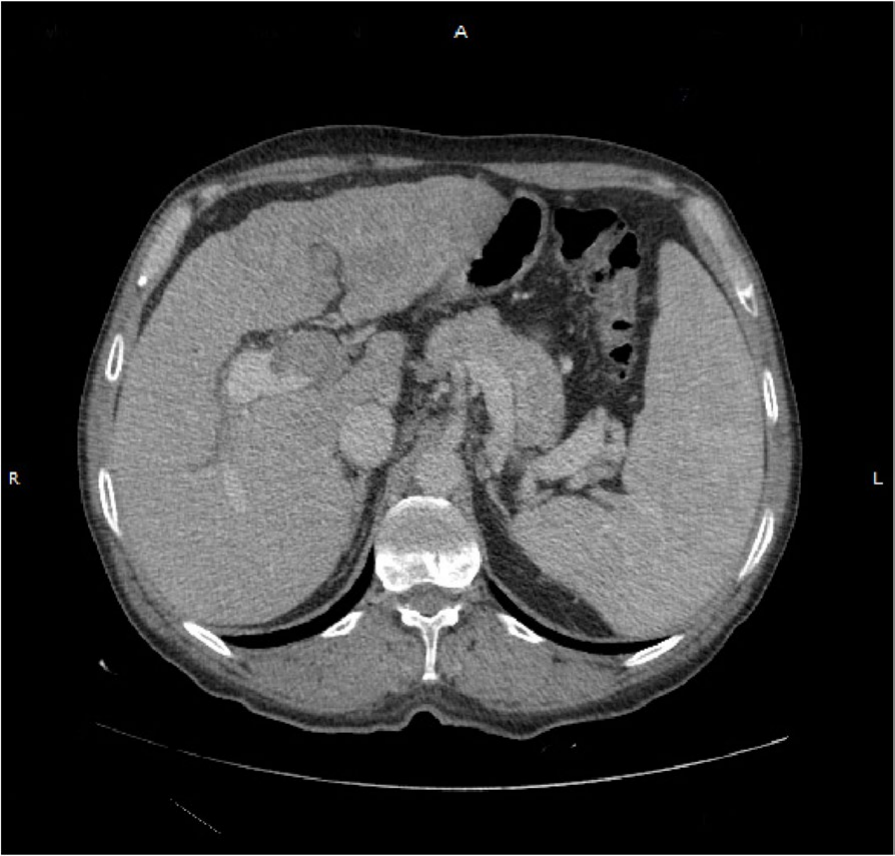
Portal phase of CT abdomen showing washout with infiltration of portal vein, with near total thrombus, which appears the same in the delayed phase

**Fig. 4:**
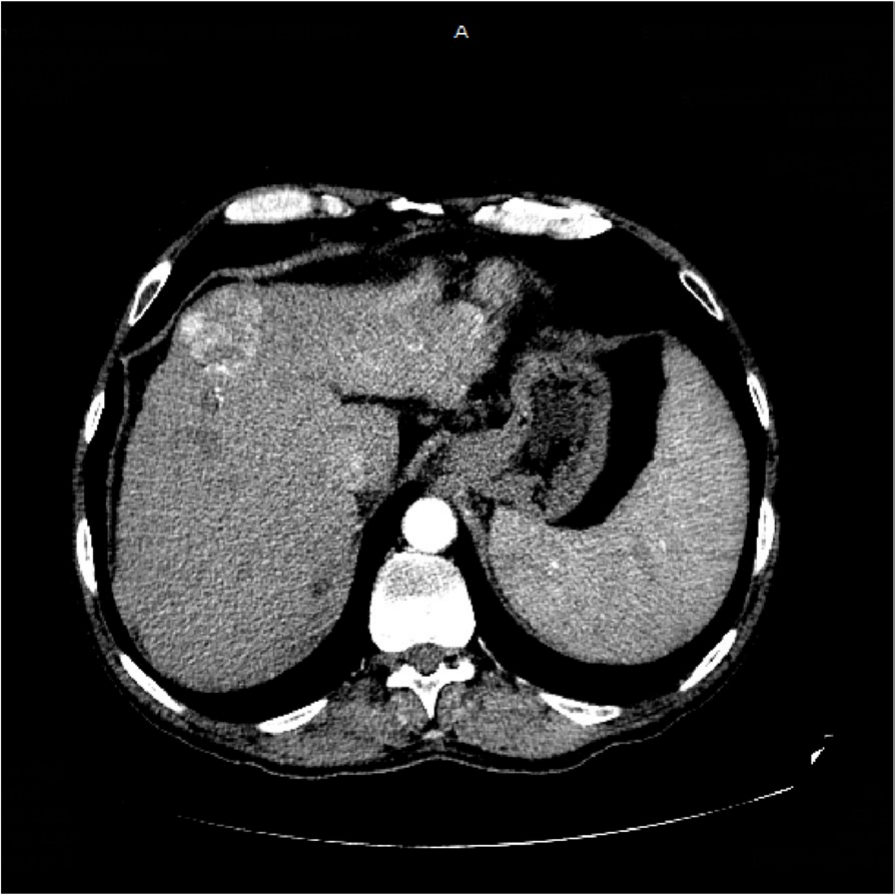
Another arterial phase showing well defined focal lesion in segment 4a measures 4.3 × 4.3 cm show enhancement

Multi-slice CT scan of the abdomen after I.V. contrast administration with triphasic liver study in 7/2022 (Figs. 2, 3, 4):

### Findings

The liver is of mild enlarged with cirrhotic CT texture, fairly defined focal lesion in left lobe segment II measures 13.0 × 7.3 cm show diffuse enhancement in arterial phase and washout out in portal and delayed phase with infiltration of portal vein, with near total thrombus, well defined focal lesion in segment 4a measures 4.3 × 4.3 cm show enhancement in arterial phase and washout in portal and delayed phase mostly hepatocellular carcinoma, ill-defined hypo dense non enhancing focal lesion in segment VII measures 2.0 × 2.3 cm likley of benign nature, dilated near totally thrombosed portal vein no intra-hepatic or extra-hepatic biliary radicle dilatation, mild enlarged spleen with no focal lesions seen, normal size, parenchymal thickness & density of both kidneys with no stones or back-pressure changes, normal appearance of the pancreas, aorta, I.V.C and both supra renals, no abdominal lymph node enlargement seen, neither ascites nor abdominal collections noted, clear scanned lung bases.

**Fig. 5:**
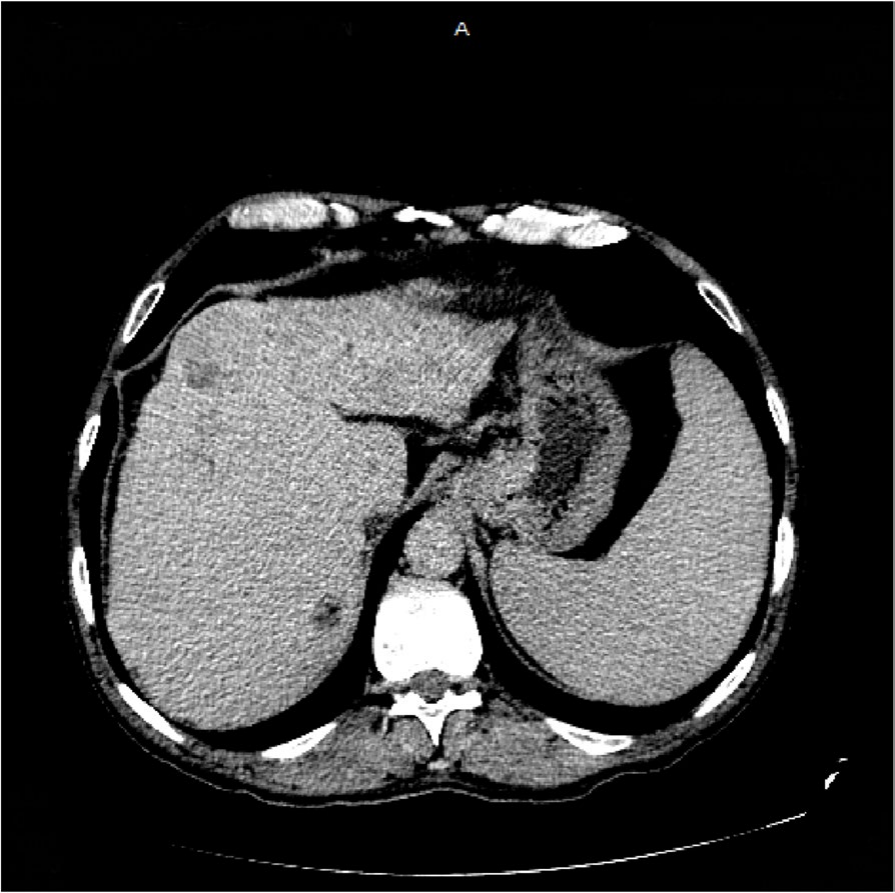
Delayed phase CT abdominal scan showing delayed washout, hypo vascular hepatocellular carcinoma

Multi-slice CT scan of the abdomen pelvis after I.V. contrast administration with triphasic liver study in 7/2023, (Fig. 5):

In comparison to the last study dated 7/2022.

Stationary course as regard left liver lobe infiltrative HCC lesion with malignant thrombosis of left PV and main PV, stationary course as regards segment VII focal lesion with faint arterial enhancement and delayed washout, hypo vascular hepatocellular carcinoma, stationary course as regards multiple enlarged malignant left Para-aortic LNs, the largest measures.

Pelvic—Abdominal Ultra Sound (US) at: 4/5/2023 showing:

The liver is mildly enlarged liver with multiple variable-sized bilobed focal lesions …multifocal HCC, dilated thrombosed left branch &main portal vein with cavernomatous transformation, moderately enlarged spleen, Para-aortic metastatic lymph nodes, 3,5 cm.

## Discussion

Of the seven reported cases of solitary HCC–skull metastasis without a primary cancer known at time of presentation except in two cases were previously identified, the average patient age at the time of diagnosis was 53.8 (range: 40–71 years) (Table 1). All patients were male which reflects the high incidence of HCC – skull metastasis in male patients. Liver disease was found in 4/7 patients (hepatitis B in one patient, long-term alcohol abuse in one patient, and previous HCC in two patients). Five patients presented with a palpable, firm bump on the head, two were associated with local pain to the scalp, and three were painless. Per Hsieh et al. 59% of patients with HCC-skull metastases reported pain at the site of skull lesion, with headaches and seizures occurring in 15% and 3% of patients, respectively [6]. If there is a skull–based lesion it presents with a neurological squeal, including right cranial nerve III, IV, and V palsy (ptosis, restricted ocular movement, and diplopia) No other patient with neurological abnormalities. Only one patient presented with the knowledge of primary liver carcinoma, and no patient presented with symptoms consistent with hepatological or gastrointestinal pathology [5]. According to the radiological features presented in the cases the tumors at the time of presentation were large (mean: 8 cm, range: 4 cm, 11 cm). It's noted that most skull metastases are hypervascular like in Trivedi et al. and Jiang et al. [7–9], osteolytic destruction of the skull with intracranial extension, but without penetration of the meninges or brain parenchyma [8, 9] and expansile [10]. On CT the tumors appeared as heterogenous lesions. All the tumors appeared as interosseous masses and didn't violate the dura or galea. According to the MRI, All cases involving the calvarium were described as homogenous, well-defined lesions with gadolinium enhancement on T1-weighted MRI. On T2 sequences, the tumors appeared isointense. After identifying the tumors by radiological maneuvers the physicians choose the clinical management by proper investigation to reach the primary site of cancer is essential in the workup of these patients and all articles mentioned this. Ferraz et al. were not specific in their workup for this [11]. Subasinghe et al. and Jiang et al. conducted abdominal CT scans [5, 9], while Trivedi and Shim mentioned “that retrograde diagnostic workup was performed to detect primary cancer,” but without mention of specific imaging modality [9, 12]. Zachary Bernstein et al. mentioned the use of non-contrast MRI [8]. Any deferential diagnosis that contains skull metastasis should be best investigated for a primary tumor through radiological modalities especially the CT of the chest, abdomen, and pelvis with or without contrast. For the treatment, 4/7 studies performed en bloc resection with craniectomy at the site of the lesion. 1/7 performed the palliative excision of the scalp but with no mention of the rationale of palliative excision versus gross total resection [7]. Conservative treatment was recommended due to the advanced stage in about 1/7 of the cases so they did not perform surgery. Two studies utilized radiotherapy: one study used postoperative adjuvant radiotherapy (radiation parameters not specified) [11] and the other utilized radiotherapy, without surgery, of 3000 cGy over 10 fractions [9]. In the four cases that underwent surgery, the dura mater was found to be intact in three of the cases and not intact in one of the cases [11]. For the final Outcomes 3/7 cases died due to liver failure in 6 months [11] 18 months [8], 4 months, [9] not due to neurological abnormalities. 1/7 patients did not report an outcome and 1/7 of the patients were alive at 9 months after diagnosis [12]. The mean survival time in a case series of 41 patients with HCC-skull metastasis (solitary or multiple) was 8.9 months, with most patients dying due to liver failure or internal bleeding before extrahepatic metastases can affect mortality (Table 2). The patient presented in this case was strictly followed in the clinic every month to prescribe his drugs and to have a lock on his investigations till he died in his home in May 2024.

**Table 1:** Demographic, clinical, and radiographic summary of all reported solitary HCC-skull metastasis without known primary HCC [6]

Authors	Patient age and gender	Prior liver disease	Clinical presentation	Image type	Image characteristics	Tumor largest dimension (cm)	Tumor size (cm)	Tumor loctation
Sherif Wael (2025)	#, M	Hepatitis C	Swelling in the head	CT	Heterogenous enhancement: well defined	5.3	5.3 × 4.9	R, Occipital
Bernstein and Cory et al. (2023) [6]	#, M	HCC	Bump on head with headache	CT	Heterogenous enhancement: well defined	5	5 × 4.5x2.2	L, parietal
Bernstein (2022)	#, M	HCC	Bump on head with headache	CT	Heterogeneous enhancement: well-defined	-	-	I, parietal
Ferraz et al. (2016) [12]	#, M	None	Bump on head with local pain	T1 MRI	Homogenous gal enhancement well-defined	#	11 × 10x5	R,frontal
Subasinghe et al. (2015) [9]	#, M	Alcohol abuse	Painless bump on head (nonmovable)	XR CT	Unavailable	#	#	Midline occipital
Jiang et al. (2014) [5]	#, M	Hepatitis B	Painless bump on head (nonmovable)	CT T1 MRI T2 MRI	Homogenous gal enhancement well-defined	5	5 × 5	R parieto-occipital
Trivedi et al. (2009) [10]	#, M	None	R 3,4 5 cranial nerve palsy (ptosis, restricted movement, diplopia of R eyeball)	T1 MRI T2 MRI	Heterogenous	-	unavailable	Sellar, parasellar, sphenoid, extending to clivus
Shim et al. (2008) [13]	#, M	None	Nonmovable mass; intermittent mild tenderness	T1 MRI T2 MRI	Homogenous gal enhancement well-defined	4	3 × 4	Midline occipital

**Table 2:** Surgical and pathology characteristics of all reported solitary HCC-skull metastasis without known primary HCC [6]

Authors	Surgery	Dura intact?	Radiotherapy in brain?	Histopathology comments	Outcome
Sherif Wael (2025)	En bloc resection	Yes	None	Epithelial membrane antigen (EMA), Cytokeratins- 7 (CK- 7), Cytokeratins–19 (CK- 19), Heppar – 1 and CD – 15 immune stains	Alive, as of May 2024
Bernstein and Cory et al. (2023) [6]	En bloc resection	Yes	None	cytokeratin AE1, AE3, hepar, and arginase- 1	Alive at 2 years after diagnosis
Bernstein (2022)	En bloc resection	Yes	None	Cytokeratin AE1, AE3, hepar, and arginase- 1	Alive at 4 months after diagnosis
Ferraz et al. (2016) [12]	En bloc resection	No	Postoperative adjuvant radiotherapy	Villin, pCEA, CD34, CK7 and CD10, and Hepatocyte	Died 6 months after diagnosis due to liver failure
Subasinghe et al. (2015) [9]	Palliative excision of scalp	Yes	None	AFP, hepar 1 staining	Unavailable
Jiang et al. (2014) [5]	En bloc resection	Yes	None	Pleiomorphic tumor cells w/eosinophilic cytoplasm, prominent nucleoli, mitosis	Died 18 months after diagnosis due to liver failure
Trivedi et al. (2009) [10]	None	NA	3,000 cGy over 10 days to skull lesion	r AFP, cytokeratin, AE1, and epithelial membrane antigen	Died 4 months after diagnosis

For the diagnosis of HCC, the NCCN made the guidelines that help in it, first, it starts with the screening program that starts with Patients at risk for HCC, and if these categories are present the physician should ask for ultrasound and alpha-pheto protein (AFP):

- Child–Pugh A or B cirrhosis, any etiology, Hepatitis B or Cd, Alcohol-associated cirrhosis, Nonalcoholic steatohepatitis, and Other etiologies.
- Child–Pugh C cirrhosis, transplant candidate. Without cirrhosis Hepatitis B

**Fig. 6:**
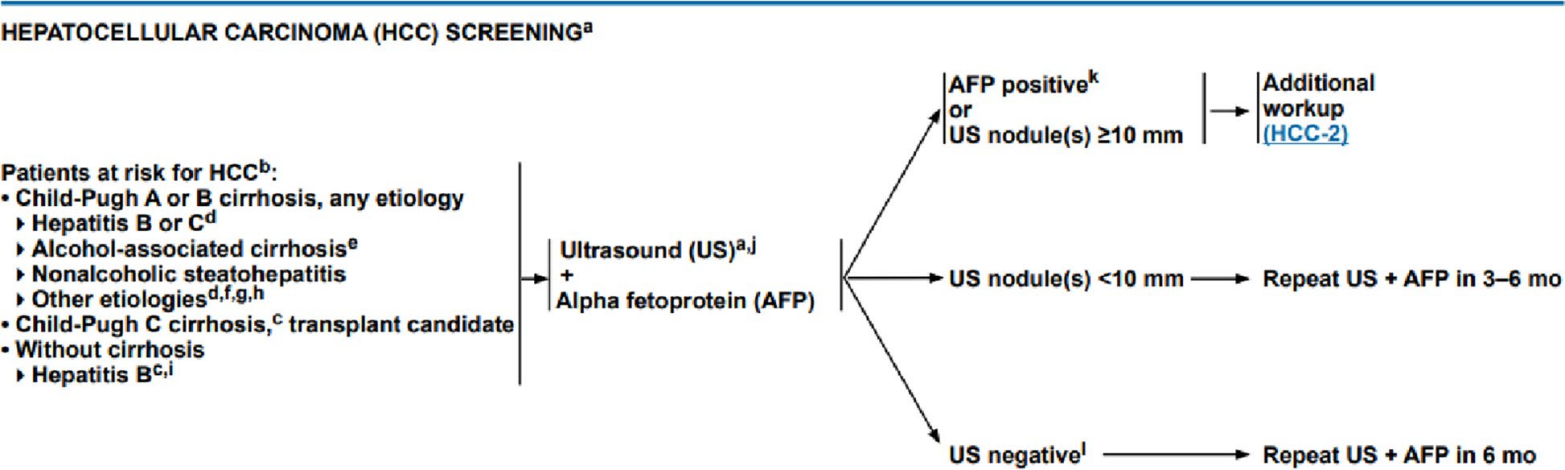
NCCN screening program for hepatocellular carcinoma [14]

**Fig. 7:**
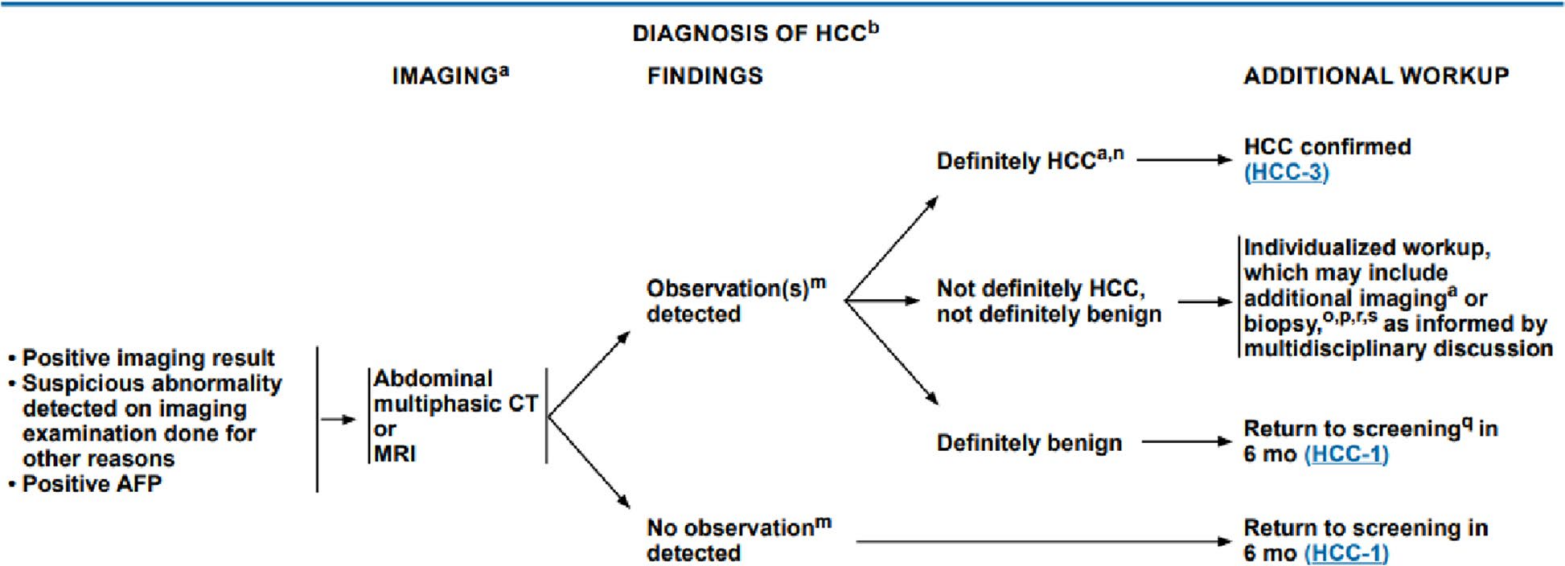
NCCN diagnosis of HCC findings [14]

If the AFP is positive or the US shows a nodule > 10 mm so needs further work. If US nodule(s) < 10 mm repeat US + AFP in 3–6 months and if US negative Repeat US + AFP in 6 months (Figs. 6 and 7).

After the screening and for the confirmation of the diagnosis some criteria should be fulfilled:

- Positive imaging result
- Suspicious abnormality detected on imaging examination done for other reasons
- Positive AFP

An abdominal CT or MRI should be done and there are two possibilities:

- Observation detected that have three ways:
  1. Definitely HCC so HCC confirmed.
  2. Not definitely HCC, not definitely benign so Individualized workup, which may include additional imaging or biopsy, as informed by multidisciplinary discussion.
  3. Definitely benign so return to screening in 6 months.

- No observation detected so return to screening in 6 months.

## Conclusion

HCC is one the most important leading causes of bone metastasis; despite the rare presentation of skull metastasis, it should remain in consideration to be one of the differential diagnoses for skull metastasis. In this case, we try to show the ways we used to diagnose and control the hepatocellular carcinoma metastasis to the skull in looking well patient and the importance of putting in mind the hepatocellular carcinoma metastasis to the skull as a deferential diagnosis for single skull lesion.

## Data Availability

No datasets were generated or analysed during the current study.

## Abbreviations

HCC: Hepatocellular Carcinoma
CT: Computed Tomography
US: Ultrasound
EMA: Epithelial membrane antigen
CK- 7: Cytokeratins- 7
CK- 19: Cytokeratins–19
AFP: Alpha-pheto protien
